# Collagen Biomarkers Quantify Fibroblast Activity In Vitro and Predict Survival in Patients with Pancreatic Ductal Adenocarcinoma

**DOI:** 10.3390/cancers14030819

**Published:** 2022-02-06

**Authors:** Neel I. Nissen, Astrid Z. Johansen, Inna Chen, Julia S. Johansen, Rasmus S. Pedersen, Carsten P. Hansen, Morten A. Karsdal, Nicholas Willumsen

**Affiliations:** 1 Biotech Research & Innovation Centre (BRIC), University of Copenhagen (UCPH), 2200 Copenhagen, Denmark; 2 Biomarkers & Research, Nordic Bioscience, 2730 Herlev, Denmark; rap@nordicbio.com (R.S.P.); mk@nordicbio.com (M.A.K.); nwi@NordicBio.com (N.W.); 3 Department of Oncology, Copenhagen University Hospital—Herlev and Gentofte, 2730 Herlev, Denmark; Astrid.Zedlitz.Johansen@regionh.dk (A.Z.J.); Inna.Chen@regionh.dk (I.C.); Julia.Sidenius.Johansen@regionh.dk (J.S.J.); 4 Department of Medicine, Copenhagen University Hospital—Herlev and Gentofte, 2730 Herlev, Denmark; 5 Department of Clinical Medicine, Faculty of Health and Medical Sciences, University of Copenhagen, 2200 Copenhagen, Denmark; 6 Department of Biomedical Science, University of Copenhagen (UCPH), 2200 Copenhagen, Denmark; 7 Department of Surgery, Copenhagen University Hospital—Rigshospitalet, 2100 Copenhagen, Denmark; carsten.palnaes.hansen@regionh.dk

**Keywords:** collagen, biomarkers, fibroblasts, CAF, fibrosis, tumor, PDAC, extracellular matrix

## Abstract

**Simple Summary:**

Pancreatic ductal adenocarcinoma (PDAC) is a devastating disease. New tools which can aid in the understanding of PDAC biology and novel drug development are needed. We established an in vitro fibroblast model in combination with collagen biomarkers as a translational anti-fibrotic drug screening tool. Furthermore, we assessed the prognostic value of the collagen biomarkers in patients with PDAC. We found that collagen biomarkers quantify fibroblast activity in vitro and predict the survival rate in patients with pancreatic ductal adenocarcinoma.

**Abstract:**

The use of novel tools to understand tumour-fibrosis in pancreatic ductal adenocarcinoma (PDAC) and novel anti-fibrotic treatments are highly needed. We established a pseudo-3D in vitro model including humane pancreatic fibroblasts (PFs) and pancreatic cancer-associated fibroblasts (CAFs) in combination with clinical collagen biomarkers, as a translational anti-fibrotic drug screening tool. Furthermore, we investigated the prognostic potential of serum collagen biomarkers in 810 patients with PDAC. PFs and CAFs were cultured in Ficoll-media. Cells were treated w/wo TGF-ß1 and the anti-fibrotic compound ALK5i. Biomarkers measuring the formation of type III (PRO-C3) and VI (PRO-C6) collagens were measured by ELISA in supernatant at days 3, 6, 9, and 12. PRO-C3 and PRO-C6, and their association with overall survival (OS), were evaluated in serum with PDAC (*n* = 810). PRO-C3 and PRO-C6 were upregulated in CAFs compared to PFs (*p* < 0.0001.). TGF-ß1 increased PRO-C3 in both PFs and CAFs (*p* < 0.0001). The anti-fibrotic compound ALK5i inhibited both PRO-C3 and PRO-C6 (*p* < 0.0001). High serum levels of PRO-C3 and PRO-C6 in patients with PDAC were associated with short OS (PRO-C3: HR = 1.48, 95%CI: 1.29–1.71, *p* < 0.0001 and PRO-C6: HR = 1.31, 95%CI: 1.14–1.50, *p* = 0.0002). PRO-C3 and PRO-C6 have the potential to be used both pre-clinically and clinically as a measure of tumor fibrosis and CAF activity.

## 1. Introduction

Pancreatic ductal adenocarcinoma (PDAC) is a lethal disease with a 5-year survival rate of 10% [1]. The incidence of PDAC is rising, and recently it was estimated that PDAC will become the second leading cause of cancer-related death by 2030 [1].

Two main reasons for the poor outcome of patients with PDAC are late diagnosis in advanced disease stages and poor efficacy of interventions [2,3]. Approximately 80% of patients present with locally advanced or metastatic disease at the time of diagnosis and are not eligible for surgery. Standard of care for these patients is limited to conventional chemotherapies, which give only minimal, or no, overall survival (OS) benefit [3,4,5,6,7].

One factor that contributes significantly to the high resistance to chemotherapy in PDAC is the extensive stromal entity in the tumor. The PDAC stroma may comprise up to 80% of the tumor mass with the major components being the extracellular matrix (ECM) as well the cancer-associated fibroblasts (CAFs) [8]. In the pancreas, most of the CAFs originate from quiescent pancreatic fibroblasts (PFs), which upon activation, by e.g., transforming growth factor receptor beta (TGF-ß), acquire a more spindle like-phenotype that express mesenchymal markers such as vimentin, alpha-smooth muscle actin (α-SMA), fibroblast specific protein-1 (FSP1), and fibroblast activating protein (FAP) [9]. CAFs possess many functions in the tumor microenvironment (TME). They secrete growth factors such as TGF-ß and vascular endothelial growth factor (VEGF), enzymes such as metalloproteinases, and lysyl oxidases and communicate with the immune system [10]. Overall, factors that are promoting tumor progression, invasion, metastasis, and immune modulation. Importantly, CAFs are the main contributor to tumor fibrosis, also called desmoplasia, through their increased synthesis of ECM proteins such as collagens, and cross-linking enzymes, creating a tumorigenic fibrotic environment surrounding the PDAC tumor [11,12,13]. Tumor fibrosis is thought to create a dense ECM-rich fibrotic cap surrounding the tumor, resulting in a hypoxic and avascular TME, making PDAC resistant to most therapies [8,14,15].

Using biomarkers in the drug development pipeline, anywhere from the translational phases to clinical trials, may provide high value and reduce time, cost, and increase the success rate of approved drugs [16,17]. During the past few years, biomarkers originating from the fibrotic TME have gained more attention [18]. We and others have shown that non-invasive biomarkers measuring ECM turnover products from fibroblast-derived collagens are increased in various cancer types and predictive of survival and treatment response [19,20,21,22,23,24,25]. As an example, PRO-C11-511, a biomarker measuring the pro-peptide of type XI collagen has shown to be increased in serum from patients with PDAC and is predictive of survival [24]. In addition, C4G, a biomarker measuring granzyme B degraded type IV collagen, assesses T-cell response and can identify patients with malignant melanoma responding to immune checkpoint inhibitors [19]. Fibrillar collagens are the most well-studied collagens, and the two fibrillar fibroblast-derived collagens, type III- and VI collagen, have shown potential as biomarkers in cancer [19,20,22,23,25,26]. Type III and VI collagens are present in most tissue within the interstitial matrix of the ECM and are augmented in many cancer diseases [20,27,28,29,30,31,32,33,34]. They have been directly linked to tumorigenesis, by being involved in cell proliferation, migration, invasion and metastasis, inflammation, and drug resistance [35]. As the amount and activity of CAFs and tumor fibrosis have been shown to be prognostic for survival in many types of cancer, several clinical trials are exploring drugs targeting CAFs, either directly, or by targeting downstream CAF signaling, such as anti-stromal therapies [36]. While CAFs and tumor fibrosis have promising potential as novel targets there are also challenges associated with this approach [37,38,39]. Preclinical models to understand the function of fibroblasts and their fibrotic activity together with supporting correlative or complementary analyses of patient material (biomarkers) may help understand this complex biology and indicate which treatment strategies should be applied to target CAFs and tumor fibrosis.

The function of fibroblasts can be studied in vitro by the scar-in-a-jar model (SiaJ). SiaJ was originally developed by Chen et al. [40] and subsequently refined by us for pulmonary and skin fibrosis modelling by prolonged culture and the addition of ECM and collagen biomarkers for applicability as a pre-clinical drug screening tool to evaluate the fibrotic component [41,42]. Inclusion of ECM and collagen biomarkers is key for translational purposes and may ultimately aid in the selection of the best-in-class anti-CAF/anti-fibrosis compounds as well as selecting which patients to treat with these compounds as a prerequisite for the optimal clinical benefits for patients.

Inspired by the SiaJ for lung and skin fibroblast, we established and validated a model including humane PFs or pancreatic CAFs, which in combination with translational collagen biomarkers of type III collagen (PRO-C3) and type VI collagen (PRO-C6) synthesis, may inform on pro- and/or anti-fibrotic effects in vitro. This might help in the understanding of modulators of tumor fibrosis and fibroblast activity and hence could have the potential to be used as a novel drug-screening tool for PDAC. Furthermore, we investigated the prognostic potential of serum PRO-C3 and PRO-C6 in patients with PDAC, to evaluate the translational value of the PDAC SiaJ model.

## 2. Materials and Methods

### 2.1. Fibroblast Cell Cultures—Scar-In-A-Jar

The SiaJ, developed by Chen et al. [40], has been suggested as a drug screening tool in pulmonary and skin fibrosis diseases [41,42]. The model is a pseudo-3D model in which fibroblasts are cultured in an in-vivo like crowded condition using macromolecules. The dense environment promotes fibroblasts to release collagen and cross-linking enzymes, which can be studied in the supernatant and at the cell plate surface.

Native human quiescent PFs and CAFs were purchased from Neuromics (cat#SC00A05 and cat#CAF08, respectively, Edina, MN, USA). PFs were cell authenticated with PCR (Actin, cytochrome B, and COX1), Karyotype and a DNA profile to confirm human cells. Pancreatic CAFs were phenotypically tested by Vitro Biopharma for markers including CD105, CD90, CD44, CD326, CD133, FAP, GFAP, FSP1, a-SMA, and Vimentin. PFs and CAFs (passage #3–4) were cultured until 80% confluency in VitroPlus III, low serum, complete purchased from Neuromics (cat# PC00B1, Edina, MN, USA). Culture flasks were coated with 5 ug/cm^2^ type I collagen purified from rat tail tendon (cat# P8188, Innoprot, Derio, Bizkaia, Spain). At 80% confluency cells were seeded in 48-well plates with 30.000 cells per well and media was changed to Gibco DMEM + GlutaMAX (cat# 31966047, Thermo Fisher Scientific, Waltham, MA, USA) supplemented with 10% fetal bovine serum (FBS) (cat# F7524, Sigma Aldrich, St. Louis, MO, USA) and 1% penicillin/streptavidin (P/S) (cat# P4333, Sigma Aldrich, USA). After 24-h (day 0) the culture media was replaced with ficoll media to culture cells in a ‘crowded’ environment with and without treatments [40]. Ficoll media consisted of; 50% Gibco DMEM + GlutaMAX supplemented 0.4% FBS, 1% P/S and 50% 70 and 400 kDa Ficoll^TM^ (cat# 17-0310-50 and 17-0300-50, GE healthcare, Chicago, IL, USA) dissolved in DMEM + GlutaMAX supplemented with 0.4% FBS, 1% P/S and L-ascorbic acid (0.05 mg/mL) (cat# A9256, Sigma Aaldrich, St. Louis, MO, USA). Cells were treated, in two to six technical replicates, with either 0 (untreated), 0.08nM TGF-ß1 (cat# 100-B-010, rndsystems, Minneapolis, MN, USA) or 1.9 μM activin receptor-kinase 5 inhibitor (ALK5i) (cat# S8822, Sigma Aaldrich, St. Louis, MO, USA). On days 3, 6, 9, and 12 cell supernatants were removed and stored at −20 °C until analysis. After removal of cell supernatant, fresh ficoll media including stimulations were added to respective wells. The experiment was repeated in three to five biological replicates.

### 2.2. Assessment of Metabolic Cell Viability by Alamar Blue

To assess metabolic activity 10% Alamar blue (cat. no. DAL1100, Invitrogen, Waltham, MA, USA), diluted in Gibco DMEM + GlutaMAX supplemented with 10% FBS and 1% P/S, was added to each well after cell supernatant removal on day 12. Cells were incubated at 37 °C until the Alamar blue solution changed color from blue to purple. The purple solution was transferred to a black 96-well plate and the colorimetric change was measured with 450 nm excitation wavelength and subtracting the background using 590 nm emission wavelength on a SpectraMac ELISA reader.

### 2.3. Decellularization of Matrix, Sirius Red Staining, and Fibril Orientation Quantification

To assess collagen orientation and alignment of the deposited matrix, cells were decellularized from their wells. On day 12, wells were washed gently with phosphate buffered saline (PBS). Next, extraction buffer (PBS + 0.5% Triton X-100 + 20 mM NH_2_OH) was added to the wells. Matrices were incubated at 37 °C until no intact cells were visualized. After detaching, PBS was added to dilute debris. Wells were stored in PBS overnight at 4 °C. After 24 h, the diluted cell debris was carefully removed. Wells were first washed with PBS and then with PBS^+^ containing 1 mM CaCl_2_ and MgSO_4_. Wells were stored at 4 °C in PBS^+^ supplemented with 1% P/S until Sirius red staining [43].

To visualize the deposited matrix, the decellularized matrices were stained with Sirius red. Decellularized wells were carefully washed with PBS. Sirius red staining solution, containing 0.5 g Sirius Red F3B + 500 mL saturated aqueous solution of picric acid, was added to each well and incubated for one hour. After incubation, wells were incubated for 2 min, first in 70% ethanol and then in 96% ethanol. The Sirius Red-stained deposited matrices were visualized under a bright field microscope at 40 x magnification. Collagen fiber orientation was analyzed using the semi-quantitative FibrilTool in ImageJ [44] (ImageJ, version 1.53n, provided by National Institutes of Health). This tool provides the average orientation and anisotropy of fibers in a given region of interest (ROI). Collagen fiber anisotropy was used as a score of fiber alignment; 0 for no order, and 1 for perfectly ordered (parallel fibers) [44]. Thus, the higher anisotropy, the more parallel fibers. Fiber anisotropy was based on mean values from 25 ROIs five wells per cell type/stimuli from Sirius Red-stained matrix pictures from PFs and CAFs. The orientation of the green line corresponds to the average orientation of fibers in the picture, whereas the lengths are proportional to the anisotropy.

### 2.4. Assessment of Collagen Formation in Cell Cultures and Human Serum Samples

To assess the formation of type III and VI collagen in the supernatant from PFs and CAFs, and type III and VI collagen in human serum samples, biomarkers reflecting the formation of these collagens (PRO-C3, and PRO-C6) were measured in cell supernatant and human serum by competitive enzyme-linked immunosorbent assays (ELISA). Biomarkers were measured according to manufactures’ procedures (Nordic Bioscience A/S, Herlev, Denmark).

### 2.5. Patients

PRO-C3 and PRO-C6 were quantified in pretreatment serum samples from 810 patients with PDAC (stage 1–4) (see Table 1 for patient demographics). All patients were included in the Danish BIOPAC study “Biomarkers in patients with pancreatic cancer” (BIOPAC, ClinicalTrials.gov.ID: NCT03311776), an open cohort multi-center study initiated in July 2008 [45]. The retrospective study (using serum samples from the BIOPAC study) “Prognostic potential of serum biomarkers reflecting tumor fibrosis (desmoplasia) and ulceration in patients with pancreas cancer” was approved in 2016. The patients were recruited from six Danish hospitals from December 2008 until September 2017. The patients were followed until 20 December 2021 or death, whichever came first. The association between PRO-C3 biomarker levels and OS in the Danish BIOPAC cohort has previously been published by Chen et al. Here patients were followed until 24 October 2018 [26]. The PDAC patients had histologically confirmed cancer and they were operated on and/or treated with different types of chemotherapy according to national guidelines). The study was carried out in accordance with the recommendations of the Danish Regional Committee on Health Research Ethics. The BIOPAC protocol was approved by the Danish Regional Committee on Health Research Ethics (VEK ref. KA-20060113; and the retrospective protocol VEK H-17039022) and the Data Protection Agency (j.nr. 2006-41-6848, 2012-58-0004, HGH-2015-027; I-Suite j. nr. 03960; and PACTIUS P-2020-834). All subjects gave written informed consent in accordance with the Declaration of Helsinki, version 8. Blood samples were obtained before the first treatment (surgery or first-line palliative chemotherapy). Samples were processed according to nationally approved standard operating procedures for blood (https://www.herlevhospital.dk/BIOPAC/Sider/default.aspx assessed on 14 January 2022)). Serum samples and clinical data from patients were collected prospectively. Serum samples were measured blinded to the clinical information. Clinical data included: age, gender, number of metastatic sites, liver metastasis, body mass index (BMI), stage (American Joint Commission on cancer, 8th edition), diabetes, tobacco use, alcohol use, carbohydrate antigen 19-9 (CA19-9), performance status (PS), Charlson age comorbidity index (CACI) and OS [45].

### 2.6. Statistical Analysis

Differences in biomarker measurements between cells and treatments were assessed using a 2way ANOVA with Sidak’s and Tukey’s multiple comparisons. Differences in cell viability between cells and treatments were assessed using a Mann-Whitney test and Kruskall-Walis tests. Differences in anisotropy were assessed using a 1way Anova with Kruskall-Wallis multiple comparisons. Biomarker results were reported in accordance with the REMARK (reporting recommendations for tumor marker prognostic study) guidelines [46]. Kaplan-Meier curves were used to assess the difference in OS between high (>median) and low (≤median) biomarker levels. A univariate Cox proportional-hazard regression model was used to calculate the hazard ratios (HR) with 95% confidence interval (Cl) for short OS per biomarker levels (continuous and >median vs. ≤median), independently and in combinations, and clinical co-variates: age (continuous), gender (female vs. male), number of metastatic sites (≥1 vs. 0), liver metastasis (yes vs. no), BMI (continuous), stage (3 + 4 vs. 1 + 2), diabetes (yes vs. no), tobacco use (ever vs. never), alcohol use (below and above the Danish Health Authority recommendations (DHAR)), CA19-9 (>median vs. ≤median (median = 506 U/mL)), PS (1 + 2 + 3 vs. 0) and CACI (≥4 vs. <4) [47]. Variables with the statistically significant association on univariate analysis were included in multivariable models. A multivariate Cox proportional-hazard regression model including PRO-C3, PRO-C6 (>median vs. ≤median), age, metastatic sites (≥1 vs. 0), liver metastasis (yes vs. no), stage (3 + 4 vs. 1 + 2), CA19-9 (>median vs. ≤median (median = 506 U/mL)) and PS (1 + 2 + 3 vs. 0) was used to evaluate potential independent prognostic value of PRO-C3 and PRO-C6 for predicting mortality risk. Spearman’s correlation coefficient was used to quantify the correlation between PRO-C3 and PRO-C6. Kaplan-Meier curves, univariate, and multivariate analyses, with the same clinical co-variates as mentioned above, were used to assess the differences in OS and prognostic value of PRO-C3 and PRO-C6 in biomarker combinations, between three groups of patients: (1) low PRO-C3 and low PRO-C6 (LL); (2) low PRO-C3 and high PRO-C6 or high PRO-C3 and low PRO-C6 (LH or HL); or (3) high PRO-C3 and high PRO-C6 (HH). A *p*-value of *p* < 0.05 was considered statistically significant. Graph design and statistical analyses were performed using GraphPad Prism Version 9.0 (GraphPad, San Diego, CA, USA) and MedCalc version 19.3 (Medcalc, Ostend, Belgium).

## 3. Results

### 3.1. Pancreatic CAFs Have Greater Fibrotic Potential Than PFs

**Figure 1:**
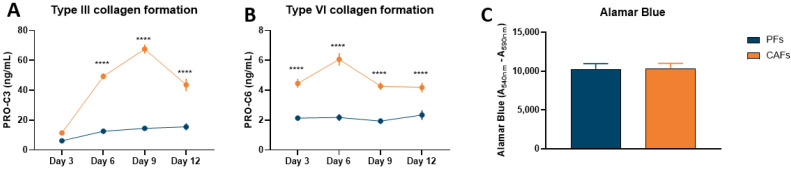
Fibrotic potential and metabolic activity in quiescent pancreatic fibroblasts (PFs) and pancreatic cancer-associated fibroblasts (CAFs). PRO-C3 (**A**) and PRO-C6 (**B**) were measured in supernatant from PFs and CAFs on day 3, 6, 9, and 12 after initiation of experiment. (**C**) On day 12 metabolic activity was assessed using Alamar Blue. *n* = 2–6 technical replicates and 3–5 biological replicates.. **** *p* < 0.0001. A *p* < 0.05 was considered significant.

To validate the PDAC SiaJ model, our initial studies examined the quantities and differences in type III and VI collagen synthesis between pancreatic CAFs and PFs. To do so, we measured PRO-C3 and PRO-C6 in supernatant from both cell types at days 3, 6, 9, and 12. On day 3, there was no difference in PRO-C3 levels between PFs and CAFs. However, on days 6, 9, and 12 there were significant increases in PRO-C3 levels in CAF supernatant compared to PFs (day 6–12: *p* < 0.0001) (Figure 1A). On day 9, PRO-C3 reached its maximum, with a 5-fold difference in type III collagen formation between CAFs and PFs (CAFs, mean: 72 ng/mL vs. PFs, mean: 14 ng/mL, *p* < 0.0001) (Figure 1A). PRO-C6 had significantly increased already on day 3 in CAF supernatant compared to PFs (day 3: *p* < 0.0001). This was also the case for days 6, 9, and 12 (day 6–12: *p* < 0.0001) (Figure 1B). Different from PRO-C3, PRO-C6 reached the highest level on day 6 with a 2-fold increase in CAF supernatant compared to PFs (CAFs, mean: 4 ng/mL vs. PFs, mean: 2 ng/mL, *p* < 0.0001) (Figure 1B). To evaluate if the differences seen in PRO-C3 and PRO-C6 between cells were a results of increased cell viability, metabolic cell viability were evaluated by Alamar Blue on day 12. There was no significant difference in cell viability between CAFs and PFs (Figure 1C).

### 3.2. TGF-ß Induces Type III Collagen Formation, but Not Type VI Collagen Formation

**Figure 2:**
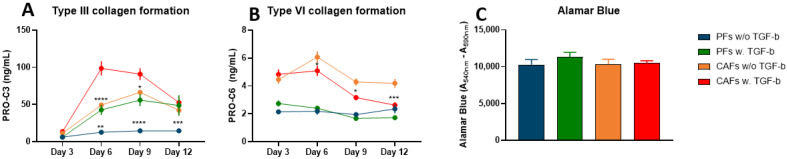
Fibrotic potential and metabolic activity in quiescent pancreatic fibroblasts (PFs) and pancreatic cancer-associated fibroblasts (CAFs) treated with TGF-ß1. PRO-C3 (**A**) and PRO-C6 (**B**) were measured in supernatant from PFs without TGF-ß1, PFs treated with TGF-ß1, CAFs without TGF-ß1 and CAFs with TGF-ß1 on day 3, 6, 9, and 12 after initiation of experiment. (**C**) At day 12 metabolic activity was assessed using Alamar Blue. *n* = 2–6 technical replicates and 3–5 biological replicates. * *p* < 0.05. ** *p* < 0.001. *** *p* < 0.001. **** *p* < 0.0001. A *p* < 0.05 was considered significant.

TGF-ß is a known stimulator of tumor fibrosis. It is involved in the transition of PFs to CAFs and in the induction of collagen synthesis in fibroblast. To further validate the PDAC SiaJ model, PFs and CAFs were treated with TGF-ß1, and the production of type III and VI collagen (PRO-C3 and PRO-C6, respectively) in response to TGF-ß1 was assessed, by measuring PRO-C3 and PRO-C6 in cell supernatant. There was no significant difference in PRO-C3 between cells treated with and without TGF-ß1 at day 3 (Figure 2A). However, from day 6–12 for PFs and day 6–9 for CAFs there were significant increases in PRO-C3 in supernatant from cells treated with TGF-ß1 compared to no treatment (Figure 2A). At day 6, there was a 2-fold increase in PRO-C3 between CAFs treated with TGF-ß1 and no treatment (CAFs w/o TGF-ß1, mean = 49 ng/mL vs. CAFs w. TGF-ß1, mean = 99 ng/mL, *p* < 0.0001) (Figure 2A). At day 9, there was a 4-fold increase in PRO-C3 between PFs treated with TGF-ß1 and no treatment (PFs w/o TGF-ß1, mean = 15 ng/mL vs. PFs w. TGF-ß1, mean = 56 ng/mL, *p* < 0.0001) (Figure 2A). Interestingly, on days 6–12, PFs treated with TGF-ß1, reached the levels of PRO-C3 measured in supernatant from untreated CAFs (Figure 2A). Opposite PRO-C3, TGF-ß1 did not induce a PRO-C6 increase in PFs (Figure 2B). Furthermore, PRO-C6 was significantly decreased in CAF supernatant at day 3–12, meaning that type VI collagen production in CAFs might be inhibited by TGF-ß1 (Figure 2B). Again, no significant differences in cell viability were observed between cell types and treatments (Figure 2C).

### 3.3. Type III and VI Collagen Production Is Inhibited by ALK5i in CAFs

**Figure 3:**
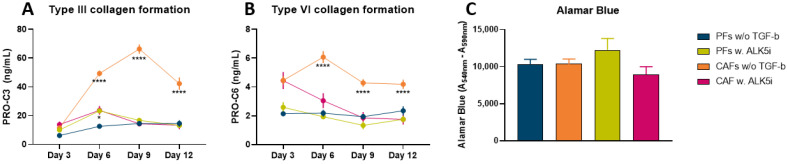
Fibrotic potential and metabolic activity in quiescent pancreatic fibroblasts (PFs) and pancreatic cancer-associated fibroblasts (CAFs) treated with the TGF-ß1 inhibitor activin receptor-kinase 5 inhibitor (ALK5i). PRO-C3 (**A**) and PRO-C6 (**B**) were measured in supernatant from PFs without TGF-ß1, PFs treated with ALK5i, CAFs without TGF-ß1 and CAFs with ALK5i on day 3, 6, 9 and 12 after initiation of experiment. (**C**) On day 12 metabolic activity was assessed using Alamar Blue. *n* = 2–6 technical replicates and 3–5 biological replicates.. **** *p* < 0.0001. A *p* < 0.05 was considered significant.

Anti-TGF-ß compounds are highly investigated in the clinic and several clinical trials are exploring this class of compounds as potential drug candidates [48,49,50]. To evaluate the PDAC SiaJ model’s potential as a drug-screening tool, PFs and CAFs were treated with an anti-TGF-ß inhibitor, ALK-5i. ALK-5 is a type I receptor of the TGF-ß superfamily (also called TGF-ß1 receptor (TGFßR1)), which TGF-ß binds to with high affinity. ALK-5i did not affect PRO-C3 or PRO-C6 in PFs supernatant which remained at baseline levels (Figure 3A and 3B). However, both markers were significantly decreased in supernatant from CAFs on days 6–12. On day 9, there was a significant 5-fold decrease in PRO-C3 in CAFs treated with ALK5i compared to untreated CAFs (CAFs w/o TGF-ß1, mean = 66 ng/mL vs. CAFs w. ALK5i, mean = 14 ng/mL, *p* < 0.0001) (Figure 3A). Likewise, at day 6 there was a 2-fold decrease in PRO-C6 in CAFs treated with ALK5i compared to untreated CAFs (CAFs w/o TGF-ß1, mean = 6 ng/mL vs. CAFs w. ALK5i, mean = 3 ng/mL, *p* < 0.0001) (Figure 3B). Interestingly, on day 9, PRO-C3 and PRO-C6 measured in supernatant from CAFs treated with ALK5i reached the same levels measured in supernatant from PFs without treatment (baseline levels). Finally, there were no differences in cell viability between cells types and treatments (Figure 3C).

### 3.4. Collagen Fibers from CAFs Are More Aligned Than Collagen Fibers Produced by PFs

**Figure 4:**
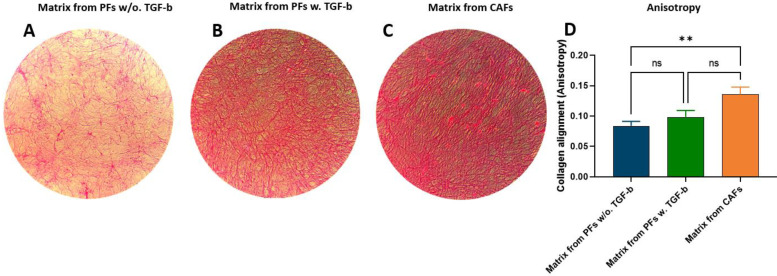
Collagen fiber alignment and orientation. Culture dishes from quiescent pancreatic fibroblasts (PFs) (**A**), PFs treated with TGF-ß1 (**B**) and pancreatic cancer-associated fibroblasts (CAFs) (**C**) was decellularized, and the deposited collagen were visualized by Sirius Red using a bright field microscope at 40× magnification. (**D**) Collagen fiber alignment, anisotropy, was analyzed using FibrilTool in ImageJ. Fiber anisotropy was used as a score of fiber alignment; 0 for no order, and 1 for perfectly ordered (parallel fibers) [44]. Thus, the higher anisotropy, the more parallel fibers. The mean is based on 25 regions of interest in 5 wells per cell type/stimuli (Figure S1: Decellularized matrices). Ns: not significant. ** *p* < 0.001. A *p* < 0.05 was considered significant.

Collagen fiber alignment and crosslinking, orchestrated by CAF, promote tumor aggressiveness and treatment resistance [12,51]. Therefore, we wished to investigate the overall collagen organization and structure in deposited matrices between PFs and CAFs. We did this by decellularizing the deposited matrices followed by Sirius red staining and visualization using a bright field microscopy at 40× magnification. There was a clear increase in Sirius Red collagen staining in CAFs compared to PFs (Figure 4A,C). This was also evident in matrices from PFs treated with TGF-ß1 compared to non-treated PFs (Figure 4A,B). In addition, CAF Sirius Red collagen staining appeared more linearized compared to the curlier matrix in PFs (Figure 4A,C). In support, using the semiquantitative FibrilTool in ImageJ, the fiber anisotropy of CAFs was significantly higher for PFs, suggesting that CAF fiber alignment is more parallel than PF fiber alignment (mean anisotropy PFs: 0.084, mean anisotropy PFs w. TGF-ß1: 0.099, mean anisotropy CAFs: 0.136 (*p* < 0.001) (Figure 4D, also represented by the length of the green line, Figure S1: Decellularized matrices). The mean orientation of the fibers in PFs matrix seemed more vertical compared to more horizontal fibers in CAF matrix (represented by the orientation of the green line, Figure S1: Decellularized matrices).

### 3.5. PRO-C3 and PRO-C6 Are Prognostic for OS in PDAC—Translational Value of the PDAC SiaJ Model

High levels of biomarkers measuring collagen fragments have been associated with poor outcomes in patients with cancer. PRO-C3 and PRO-C6 are high in supernatant from CAFs, therefore, to evaluate the translational value of the PDAC SiaJ model, we investigated the prognostic potential of PRO-C3 and PRO-C6 in patients with PDAC.

### 3.6. High Serum PRO-C3 Levels Are Predictive of Short OS in Patients with PDAC

**Figure 5:**
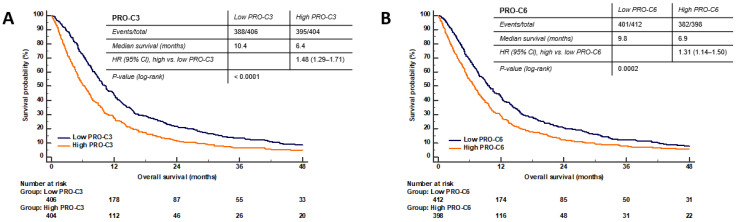
Kaplan-Meier plots showing the association between PRO-C3 (**A**), PRO-6 (**B**) and overall survival in patients with pancreatic ductal adenocarcinoma (stages 1–4, *n* = 810). Blue line: low biomarker levels (≤median). Orange line: High biomarker levels (>median). Hazard ratios (HR), 95% confidence interval (CI) and log-rank tests are shown. A *p* < 0.05 was considered significant.

Recently, Chen et al. showed that high serum levels of PRO-C3 were associated with short OS in the Danish BIOPAC cohort [26]. Since then, the clinical data and follow-up time have been updated. Here we reanalyzed PRO-C3 in the Danish BIOPAC cohort to evaluate if PRO-C3 was predictive of survival with a median follow-up time of 7.8 months. We also dichotomized patients differently than Chen et al. which used a continuous scale (per 100 ng/mL increase) to evaluate the ability of PRO-C3 to predict the OS outcome. Patients with PDAC (*n* = 810) were divided into two groups based on high and low biomarker levels (>median vs. ≤median). Patients with high PRO-C3 (>median) had a median OS at 6.4 months compared to 10.4 months for patients with low PRO-C3 levels (≤median) (log-rank *p* < 0.0001), confirming that high PRO-C3 levels are associated with shorter survival (Figure 5A). A univariate Cox analysis showed that patients with high PRO-C3 levels (>median) had a 48% increased risk of mortality compared to patients with low PRO-C3 levels (HR = 1.48, 95%CI 1.29–1.71, *p* < 0.0001) (Figure 5A and Table 2).

### 3.7. High Serum PRO-C6 Levels Are Predictive of Short OS in Patients with PDAC

Like PRO-C3, we evaluated the prognostic potential of PRO-C6 in serum from patients included in the Danish BIOPAC cohort. Patients (*n* = 810) were divided into two groups based on high and low biomarker levels (>median vs. ≤median). Patients with high PRO-C6 (>median) had a median OS at 6.9 months compared to 9.8 months for patients with low PRO-C6 (≤median) (log-rank *p* = 0.0002), confirming that high PRO-C6 levels are associated with shorter survival (Figure 5B). A univariate Cox analysis showed that patients with high PRO-C6 levels (>median) had a 31% increased risk of mortality compared to patients with low PRO-C6 levels (HR = 1.31, 95%CI 1.14–1.50, *p* = 0.0002) (Figure 5B and Table 2).

When adjusting for age, the number of metastatic sites, liver metastasis, stage CA19–9, and PS by multivariate Cox analysis, high serum levels of PRO-C3, but not PRO-C6, were significantly associated with short OS (PRO-C3: HR = 1.24, 95%CI 1.04–1.47, *p* = 0.0149. PRO-C6: HR = 1.15, 95%CI 0.97–1.36, *p* = 0.1139) (Table 2).

### 3.8. Combination PRO-C3 and PRO-C6 Are Complementary

**Figure 6:**
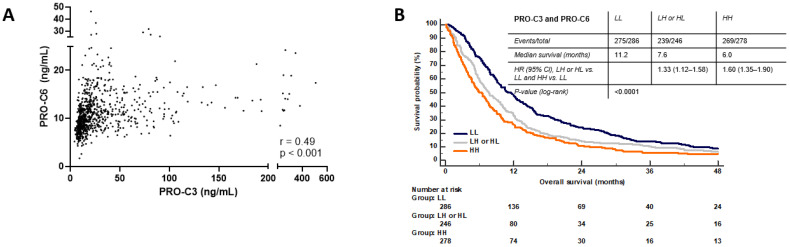
(**A**) Correlation between PRO-C3 and PRO-C6 biomarker levels were assessed using Spearman’s correlation coefficient (r). (**B**) Kaplan-Meier plots showing the association between combination PRO-C3 and PRO-6 and overall survival in patients with pancreatic ductal adenocarcinoma (stages 1–4, *n* = 810). Patients were divided in three groups: (1) low PRO-C3 and low PRO-C6 (LL), (2) low PRO-C3 and high PRO-C6 or high PRO-C3 and low PRO-C6 (LH or HL), (3) high PRO-C3 and high PRO-C6 (HH). Blue line: low biomarker levels (≤median). Orange line: High biomarker levels (>median). Hazard ratios (HR), 95% confidence interval (CI) and log-rank tests are shown. A *p* < 0.05 was considered significant.

High levels of PRO-C3 and PRO-C6 were both individually predictive of short OS and not dependent on each other. Therefore, we correlated the two biomarkers using a non-parametric Spearman’s correlation coefficient. There was a significant but weak correlation between the two biomarkers (r = 0.49, *p* < 0.001) (Figure 6A). Since there was only a weak correlation between the markers, we combined the markers to evaluate the prognostic value of having high and low levels in both biomarkers compared to having high in only one biomarker or low in both biomarkers. Patients (*n* = 810) were divided into three groups: (1) high PRO-C3 and high PRO-C6 (HH) (*n* = 278), (2) low PRO-C3 and low PRO-C6 (LL) (*n* = 286), (3) low/high PRO-C3 and low/high PRO-C6 (LH or HL) (*n* = 246). HH and LH or HL patients had a median OS at 6.0 and 7.6 months, respectively, compared to 11.2 months for patients with both low PRO-C3 and low PRO-C6 (LL) (log-rank test *p* < 0.0001) (Figure 6B). Thus, LL patients lived almost twice as long as HH patients. A univariate analysis showed that HH patients had a 60% increased risk of mortality compared to LL patients (HR = 1.60, 95%CI 1.35–1.90, *p* < 0.0001) (Table 2). Furthermore, LH or HL patients had a 33% increased risk of mortality compared to LL patient (HR = 1.33, 95%CI 1.12–1.58 *p* = 0.0014). When adjusting for age, number of metastatic sites, liver metastasis, stage, CA19-9, and PS by multivariate Cox analysis, the HH group, but not the LH or HL group, was significantly associated with short OS (HH: HR = 1.42, 95%CI 1.18–1.71, *p* = 0.0002. LH or HL: HR = 1.19, 95%CI 0.99–1.43 *p* = 0.0703) (Table 2).

## 4. Discussion

In this study, we established an in vitro model (SiaJ), using pancreatic CAFs and PFs, and evaluated its potential as a pre-clinical tumor fibrosis model in PDAC in combination with translational collagen biomarkers. Furthermore, we took the same biomarkers into the clinic by evaluating the prognostic potential in patients with PDAC.

We saw that CAFs produce higher quantities of type III and VI collagen (PRO-C3 and PRO-6, respectively) compared to PFs. In addition, when stimulated by TGF-ß1, type III collagen (PRO-C3) production was increased in PFs and CAFs. However, this was not evident for type VI collagen. When adding an anti-TGF-ß compound (ALK5i) to the cultures, both type III and type VI collagen production was decreased in CAFs. Lastly, we saw that high levels of type III and VI collagen biomarkers PRO-C3 and PRO-C6 were independent prognostic factors for short OS in patients with PDAC, and that combining the two biomarkers complemented each other, and patients with high serum levels of both PRO-C3 and PRO-C6 had the worst prognosis. Altogether, these data indicate that the PDAC SiaJ has the potential to be used as a screening model for anti-tumor fibrosis drugs, and in combination with translational collagen biomarkers can assess the direct effect on patient outcome.

Fibroblasts cultured in classic media conditions deposit collagen very slowly and in low quantities [52]. Here we cultured fibroblasts in a crowded pseudo-3D environment using ficoll. With the use of macromolecules such as ficoll the collagen deposition is accelerated and the quantity can be increased many folds [40,52]. A proper in vivo like collagen deposition is needed when screening anti-tumor fibrosis compounds to increase the chance of translational value. Both dextran sulfate and ficoll have been used as crowding macromolecules, but by the use of ficoll the collagen deposition achieves a more cross-linked collagen meshwork resembling a more in vivo like condition [40].

In this study, we saw that CAFs produce higher quantities of type III (PRO-C3) and VI collagen (PRO-C6) than PFs independent of the metabolic activity. The increases in PRO-C3 and PRO-C6 were supported by an increased deposition of collagen in the wells quantified by Sirius red. Type III and type VI collagen are fibroblast-derived collagens and known to play key roles in fibrosis and cancer. Type III collagen is involved in both proliferation and migration in pancreas cancer cell lines. Menke et al., have shown that type III collagen regulates migration in these cell lines by downregulation of E-cadherin [53]. In addition, type VI collagen is involved in key tumor-promoting processes [33,54,55,56,57,58]. The α3 chain of type VI collagen is especially involved in cancer being highly expressed in PDAC among other cancer types [59]. In tissue from patients with PDAC, the α3 chain of type VI collagen is highly expressed near tumor cells and fibroblasts [60]. Interestingly, the PRO-C6 biomarker targets the α3 chain of type VI collagen. Thus, PRO-C6 is a highly relevant biomarker to measure in PDAC.

TGF-ß is a major inducer of tumor fibrosis and has previously been shown to upregulate collagen deposition in vitro [10,41,42]. In this study, we saw a 4- and 2-fold increase in type III collagen synthesis (PRO-C3) in PFs and CAFs, respectively, when treated by TGF-ß1. In detail, PF stimulated with TGF-ß1 reached PRO-C3 levels similar to those found intrinsically in unstimulated CAFs indicating that PFs stimulated with TGF-ß1 may be more pro-fibrotic and support a myofibroblast phenotype. These data show that collagen deposition in the PDAC SiaJ model resembles what has earlier been described in the literature in in vitro models [61]. TGF-ß has also been shown to be part of the PF to CAF transition [36]. Since the PFs resemble the CAF type III collagen production after stimulation by TGF-ß1, it could be interesting to investigate if the PFs achieved a more CAF-like or myofibroblast phenotype. In contrast to the induction of type III collagen synthesis, TGF-ß1 did not stimulate type VI collagen formation (PRO-C6). Again, this was independent of the metabolic activity of the cells. It is well known that both collagens are produced by myofibroblasts and CAFs, however, the complexity of the underlying drivers and mechanism is still not fully understood. The data indicate that the synthesis of different collagens can be induced by various stimuli and that different collagens may reflect different upstream signaling processes.

While it is important to have tools to aid in understanding what drives fibroblast/CAF activity and synthesis of specific collagens, it is equally important to have tools to investigate potential anti-CAF and/or anti-fibrotic effects. Anti-TGF-ß compounds are highly investigated in the clinic and several clinical trials are exploring this class of compounds as potential drug candidates [48,49,50]. To evaluate the PDAC SiaJ models’ potential as a drug-screening tool we treated PFs and CAFs with an anti-TGF-ß inhibitor, ALK-5i. This resulted in a decrease in CAF fibrotic activity reflected by decreased levels of PRO-C3 and PRO-C6 compared to untreated CAFs. Interestingly, type VI collagen production was decreased in CAFs, even though CAFs were not stimulated by TGF-ß1 to produce type VI collagen. One explanation could be that the ALK5i inhibits the TGFßR1, which interacts with many other ligands besides TGF-ß1, and therefore results in a more broad inhibition [62]. The TGFßR1 can form complexes with platelet-derived growth factor receptor-beta (PDGFR-ß) and CD44. Upon binding of PDGF-BB, canonical TGF-ß signaling is induced. Juhl et al. have shown that PDGF-AB stimulates PRO-C6 production in dermal fibroblasts [42]. PDGF-BB might have the same function, and if present in the CAF SiaJ system (CAFs produce PDGF [63]), its function might be inhibited by ALK5i when binding to the TGFßR1/PDGFR-ß/CD44 complex, thus, resulting in an inhibition of PRO-C6 [62]. In addition to anti-TGF-ß therapies, examples of other potentially anti-fibrotic compounds that are currently being tested are vitamin A and D analogs, angiotensin, Hedgehog inhibitors, and nab-paclitaxel [21,64,65,66,67,68,69]. Unfortunately, many anti-fibrotic treatments have failed in clinical trials, despite promising pre-clinical results. One example is the hyaluronidase inhibitor PEGPH20, which showed very promising results pre-clinically by depleting hyaluronidase and thereby increasing drug delivery in a PDAC mouse model [70]. However, the clinical trials of PEGPH20 were unfortunately terminated due to the trials not meeting their endpoints of OS in patients with PDAC. One explanation for the failure of many anti-fibrotic treatments could be the dual roles of many ECM proteins [39]. Pre-clinical studies of PDAC have shown contradicting results concerning the tumor restricting and tumor-promoting roles of tumor fibrosis [71,72]. As an example, complete depletion of αSMA positive CAFs in a transgenic mouse model of PDAC has been shown to excite tumor aggressiveness [71]. Contrary to this, normalizing CAFs, rather than removing them, has shown promising results in decreasing tumor volume and increasing survival [38,66]. The fact that CAFs, treated with ALK5i, in the SiaJ model reached PRO-C3 levels similar to those found in PF, could indicate that PRO-C3 may have the potential to reflect a normalization of the fibrotic activity of CAFs. Tumor fibrosis is extremely complex, and whether it is tumor-promoting or tumor restricting might be context-dependent. Biomarkers reflecting tumor fibrosis, such as collagen biomarkers, could be a tool to improve the understanding of tumor fibrosis and potentially aid in increasing the success of novel anti-fibrotic therapies in clinical trials. 

Chen et al. have previously shown that high serum levels of the biomarker PRO-C3, measuring the formation of type III collagen, were associated with short OS in the Danish BIOPAC study cohort [26]. In another cohort of patients with PDAC, PRO-C3 has been shown to be a predictor of treatment response to an anti-stromal compound (PEGPH20) in combination with chemotherapy [21]. Here we reanalyzed PRO-C3, together with PRO-C6, in the BIOPAC cohort, however with an updated follow-up time (nearly 3 years) and dichotomized patients differently than Chen et al. which used a continuous scale (per 100 ng/mL increase). We saw that high PRO-C3 and high PRO-C6 levels were predictive of short OS. While PRO-C6 has been shown to be increased in patients with PDAC [34], to our knowledge, we are the first to show that high serum levels of PRO-C6 are also predictive of short OS in patients with PDAC. Furthermore, the prognostic value increased when combining serum PRO-C3 and PRO-C6 in patients with PDAC, supporting the in vitro data suggesting that these two collagens may have their own unique function in the tissue, tumor development, and impact on patient outcome.

Two collagens might have their own unique function, which is supported by the fact that specific chains of collagens can have differential functions. As opposed to the α3 chain of type VI collagen, which is pro-tumorigenic, the α1 and α2 chain of type VI collagen have shown to repress proliferation, migration, and invasion in a bladder cancer cell line [73]. Furthermore, recently Martino et al. showed that the α1 and α2 chain of type VI collagen is highly expressed around dormant tumors compared to more aggressive tumors [74]. Similar to the fact that specific collagen chains and domains can have unique functions in the stroma, the structure of the collagens is equally important in tumor progression. Collagens, such as type III collagen, are more aligned in tumor stroma than in healthy stroma. The more aligned stroma is also evident around more aggressive tumors, compared to dormant ones [74]. Since CAFs have been shown to orchestrate the alignment and crosslinking of the collagen fibers, we decellularized the CAF and PF matrices, to investigate the overall collagen organization and structure in the deposited matrix between the two cell types [12,51]. Our results indicated that collagen fiber alignment is more parallel in decellularized matrices from CAFs than the decellularized matrices from PF cultures. This has also been shown in a 3D model of PDAC and in tissue from patients with PDAC [75,76]. Interestingly, when looking at the relative PRO-C6 biomarker levels between PFs, PFs treated with TGF-ß and CAFs, they follow the anisotropy of the same cells. Thus, the more PRO-C6 the higher collagen alignment (anisotropy). Therefore, PRO-C6 may be a measure of collagen alignment (anisotropy). Clinically, collagen fiber alignment is a negative prognostic factor in PDAC [12,77]. This might be due to the fact that fiber alignment promotes migration, by being highways for cancer cells to travel on, and furthermore, highly aligned fibers result in stiff tissue, which increases interstitial fluid pressure, resulting in reduced drug uptake and thereby treatment resistantcy [78,79,80]. This again supports that different collagens, their chains, and structures, might have unique roles in tumor progression. Thus, it could be discussed whether “good” and “bad” collagens actually exist, as also suggested by Karsdal et al. [81]. From a drug development perspective, these differences are highly important to take into consideration when finding novel targets. From a biomarker perspective, future studies should explore the biomarker potential of different collagens, individually and in combinations.

Several limiting factors and perspectives should be noted for this study. Firstly, it is well-known that many subtypes of CAFs exist, which might bias the results presented in this study [82]. Öhlund et al., have identified two groups of CAFs called iCAFs and myCAFs in PDAC. MyCAFs are more involved in matrix deposition, whereas iCAFs are thought to play a larger role in inflammatory infiltration [83]. Other subpopulations, such as pan-CAF, apCAF, mCAF, cCAF, CAF-cluster1/2/3, have also been identified, all with different roles and tumorigenicity [82,84,85,86]. The SiaJ model might have the potential to culture different subtypes, and thereby assess novel-anti CAF/stromal compounds’ effect on CAF subtypes. The many subtypes of CAFs, and the fact that no specific CAF markers exist, complicate strategies for CAF targeting drugs. It could be discussed if targeting CAF downstream signaling might be a more relevant avenue to pursue than targeting a specific CAF subpopulation, as total depletion of CAFs has been shown to promote tumor aggressiveness [71]. Next, PFs and CAFs used in this study were not patient-matched, therefore using CAFs from different patient donors would have been desirable. Prospectively, it would be interesting to investigate the PDAC SiaJ in a co-culture system, as the interaction between cancer cells, immune cells, and fibroblasts is extremely important and could influence different treatment strategies [87,88]. In addition, culture cells in other ECM-specific substrates might influence cell phenotype [89]. Clinically, we retrospectively analyzed serum PRO-C3 and PRO-C6 in the BIOPAC study (a multicenter open cohort study where blood samples are collected prospectively before and during treatment). Therefore, the presented data should be validated in larger prospective cohorts. Furthermore, enrolled patients typically had a better PS, than patients not enrolled, and therefore were more likely to receive chemotherapy [26]. Only 20% of all registered Danish patients with PDAC were included in the BIOPAC study resulting in potential selection bias.

**Figure 7:**
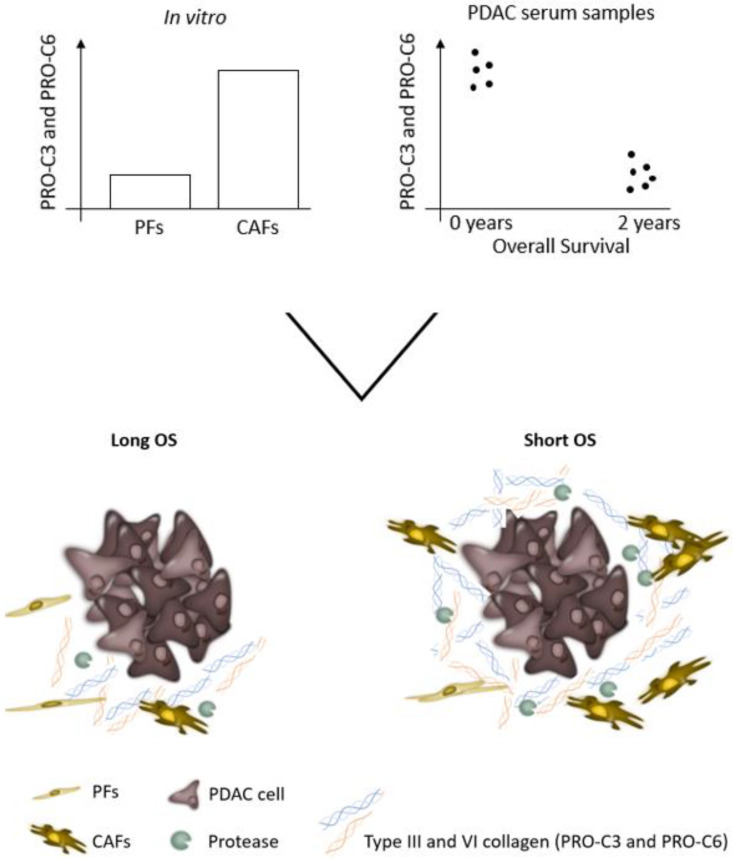
Summary figure. PRO-C3 and PRO-C6 are increased in pancreatic cancer-associated fibroblasts (CAFs) compared to pancreatic quiescent fibroblasts (PFs). High levels of PRO-C3 and PRO-C6 are prognostic for short overall survival in patients with pancreatic ductal adenocarcinoma (PDAC). This suggest that PRO-C3 and PRO-C6 can be used both pre-clinically and clinically as a measure of tumor fibrosis and CAF activity, and thereby predict survival in patients with PDAC. OS: overall survival. PRO-C3: biomarker measuring formation of type III collagen. PRO-C6: biomarker measuring formation of type VI collagen.

In this study, we showed that PRO-C3 and PRO-C6 can be used both pre-clinically and clinically. PRO-C3 and PRO-C6 have mostly been used as biomarkers in patient serum and plasma. We also showed that they can be used translationally in a cell culture model as a tool to investigate tumor fibrosis and CAF activity, as well as the effect of an anti-fibrotic drug on collagen production. Furthermore, we demonstrated clinically that PDAC patients with high biomarker levels have a short OS. Thus, it could be suggested if the high biomarker levels measured in patients reflect heightened tumor fibrosis and CAF activity resulting in poor patient prognosis (summarized in Figure 7). Bringing the same biomarkers used pre-clinically into clinical studies, during the drug development pipeline, may be an advantage in the interpretation of data. Biomarkers used across all phases of drug development may improve the success rate for approved cancer drugs [90]. Besides prognostic value in patients with PDAC, PRO-C3 and PRO-C6 might have the potential to be used as a measure of drug response in clinical trials.

In summary, we have developed and validated a pancreatic fibroblast in vitro model that can be combined with non-invasive collagen biomarkers and used as a translational anti-fibrotic drug-screening tool for PDAC. Thus, the PDAC SiaJ model, in combination with collagen biomarkers, might have the potential to be used as a preclinical anti-fibrosis drug screening model in PDAC. Furthermore, collagen biomarkers, such as PRO-C3 and PRO-C6, can be used preclinically (alone or in conjunction with the SiaJ model) and clinically as a measurement of patient outcome, and CAF and fibrosis activity, throughout the drug-development stages. 

## Figures and Tables

**Table 1:** Patient demographics.

Clinical Variables (PDAC)	Study Population (*n* = 810)
Age, (years)	
*Median (min, max)*	66 (37–89)
Gender, *n* (%)	
*Male*	433 (53%)
*Female*	377 (47%)
Number of metastatic sites, *n* (%)	
*0 site*	376 (46%)
*≥1 site*	434 (54%)
Liver metastasis (of all patients with metastasis, *n* = 434), *n* (%)	
*Yes*	331 (76%)
*No*	103 (24%)
BMI	
*Median (min, max)*	23 (14–39)
Stage	
*1*	15 (2%)
*2*	123 (15%)
*3*	237 (29%)
*4*	431 (53%)
*Unknown*	4 (<1 %)
Diabetes	
*Yes*	198 (24%)
*No*	603 (74%)
*Unknown*	9 (1%)
Tobacco	
*Ever*	484 (60%)
*Never*	251 (31%)
*Unknown*	75 (9%)
Alcohol	
*<DHAR*	554 (68%)
*>DHAR*	179 (22%)
*Unknown*	77 (10%)
CA19-9 (U/mL)	
*≤median (≤506 U/mL)*	395 (49%)
*>median (>506 U/mL)*	387 (48%)
*Unknown*	28 (3%)
Performance status, n (%)	
*0*	294 (36%)
*1*	335 (41%)
*2*	89 (11%)
*3*	5 (<1%)
*Unknown*	87 (11%)
The Charlson age comorbidity index	
*<4*	538 (66%)
*≥4*	258 (32%)
*Unknown*	14 (2%)

**Table 2:** Association between biomarker levels, clinical covariates and outcomes for patients with pancreatic adenocarcinoma (PDAC), stage 1–4. Uni- and multivariate Cox regression analyses were used to calculate the hazard ratios (HR) with 95% Cl and *p*-values. A *p* < 0.05 was considered significant. * kombi: combination of PRO-C3 and PRO-C6 in three groups: (1) low PRO-C3 and low PRO-C6 (LL); (2) low PRO-C3 and high PRO-C6 or high PRO-C3 and low PRO-C6 (LH or HL); and (3) high PRO-C3 and high PRO-C6 (HH). Abbreviations: BMI, body mass index; CACI, Charlson age comorbidity index, CA19-9, carbohydrate antigen (U/mL); DHAR, Danish Health Authority recommendations on alcohol consumption; No, number; PDAC, pancreatic ductal adenocarcinoma; PS, performance status.

Table 2: Uni- and Multivariate Analysis (Overall Survival), *n* = 810		Univariate		Multivariate		Multivariate * Kombi	
Variables		HR (95% Cl)	*p*-Value	HR (95% Cl)	*p*-Value	HR (95% Cl)	*p*-Value
PRO-C3	Continuous	1.00 (1.00–1.00)	0.0036	-	-	-	-
	>median vs. ≤median	1.48 (1.29–1.71)	<0.0001	1.24 (1.04–1.47)	0.0149	-	-
PRO-C6	Continuous	1.05 (1.03–1.07)	<0.0001	-	-	-	-
	>median vs. ≤median	1.31 (1.14–1.50)	0.0002	1.15 (0.97–1.36)	0.1139	-	-
PRO-C3 and PRO-C6	High + high vs. low + lowLow + high or high + low vs. low + low	1.60 (1.35–1.90) 1.33 (1.12–1.58)	<0.0001 0.0014	-	-	1.42 (1.18–1.71) 1.19 (0.99–1.43)	0.0002 0.0703
Age	Per year increase	1.01 (1.00–1.02)	0.0199	1.01 (1.00–1.36)	0.1694	1.01 (1.00–1.02)	0.1916
Gender	Female vs. male	0.97 (0.85–1.12)	0.7063	-	-	-	-
Number of metastatic sites	≥1 vs. 0	2.56 (2.21–2.97)	<0.0001	1.52 (1.18–2.00)	0.0011	1.51 (1.18–1.95)	0.0013
Liver metastasis	Yes vs. no	2.37 (2.05–2.75)	<0.0001	1.28 (1.01–1.62)	0.0396	1.28 (1.02–1.63)	0.0363
BMI	Continuous	0.99 (0.97–1.01)	0.2344	-	-	-	-
Stage	3 + 4 vs. 1 + 2	2.85 (2.33–3.50)	<0.0001	1.97 (1.52–2.55)	<0.0001	1.97 (1.52–2.56)	<0.0001
Diabetes	Yes vs. no	1.08 (0.92–1.27)	0.3431	-	-	-	-
Tobacco	Ever vs. never	1.06 (0.91–1.24)	0.4448	-	-	-	-
Alcohol	*>DHAR* vs. *<DHAR*	1.05 (0.88–1.24)	0.6112	-	-	-	-
CA19-9	>median vs. ≤ median	2.01 (1.69–2.39)	<0.0001	1.53 (1.30–1.80)	<0.0001	1.54 (1.23–1.70)	<0.0001
PS	1 + 2+3 vs. 0	1.58 (1.36–1.85)	<0.0001	1.45 (1.24–1.71)	<0.0001	1.44 (1.23–1.69)	<0.0001
CACI	High (≥4 vs. <4)	1.14 (0.98–1.33)	0.0860	-	-	-	-

## Data Availability

The datasets used and/or analysed during the current study are available from the corresponding author on reasonable request.

## Supplementary Materials

The following supporting information can be downloaded at: https://www.mdpi.com/article/10.3390/cancers14030819/s1. Supplementary Figure S1: Decellularized matrices from pancreatic fibroblasts (PFs) (A), PFs treated with TGF-ß1 (B) and pancreatic cancer-associated fibroblasts (CAFs) (C) stained with Sirius Red, and visualized under bright field microscopy at 40× magnification.
