# Supplementary material for: Collagen Biomarkers Quantify Fibroblast Activity In Vitro and Predict Survival in Patients with Pancreatic Ductal Adenocarcinoma
Source: Cancers (Basel). 2022 Feb 6;14(3):819. doi: 10.3390/cancers14030819 (PMC8833921; doi:10.3390/cancers14030819)
Supplement: Supplementary file 1 [file cancers-14-00819-s001.zip › cancers-1577343-supplementary.pdf]

Supplementary Materials:

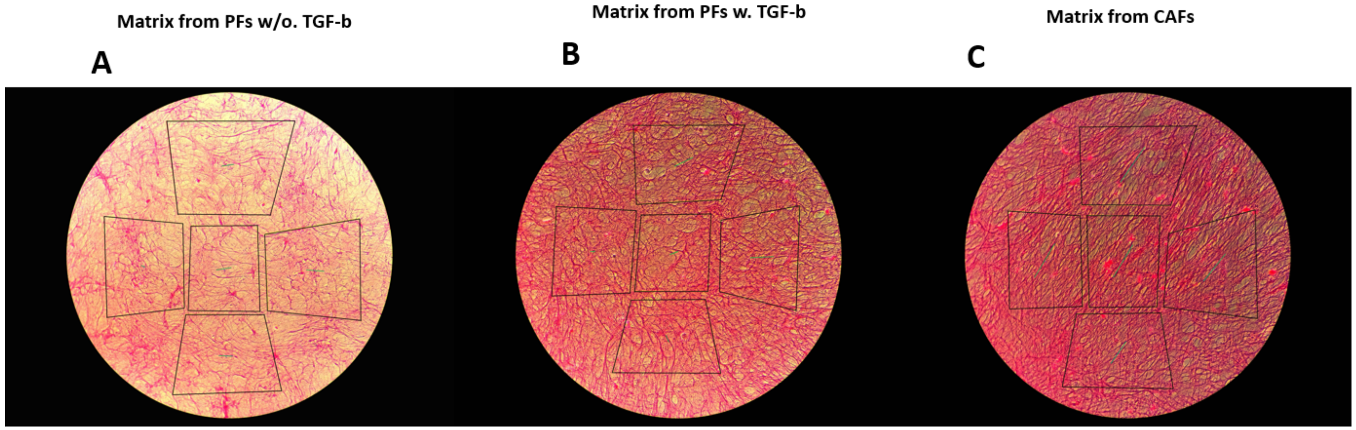

Figure S1. Supplementary figure 1: Decellularized matrices from pancreatic fibroblasts (PFs) (**A**), PFs treated with TGF-ß1 (**B**) and pancreatic cancer-associated fibroblasts (CAFs) (**C**) stained with Sirius Red, and visualized under bright field microscopy at 40 x magnification. Collagen fiber alignment and orientation, anisotropy, were analyzed using FibrilTool in ImageJ. FibrilTool analyzed the regions of interest (ROI, black squares). Fiber anisotropy was used as a score of fiber alignment; 0 for no order, and 1 for perfectly ordered (parallel fibers) [45]. Thus, the higher anisotropy, the more parallel fibers. The orientation of the green lines corresponds to the average orientation of fibers, whereas the lengths is proportional to the anisotropy. Representative pictures of one well per cell type/stimuli out of 25 ROI's in five wells.
